# Prevalence and Associated Factors of Prehypertension and Hypertension in Iranian Population: The Lifestyle Promotion Project (LPP)

**DOI:** 10.1371/journal.pone.0165264

**Published:** 2016-10-26

**Authors:** Jafar Sadegh Tabrizi, Homayoun Sadeghi-Bazargani, Mostafa Farahbakhsh, Leila Nikniaz, Zeinab Nikniaz

**Affiliations:** 1 Tabriz Health services management research center, Faculty of management and medical informatics, Tabriz University of Medical Sciences, Tabriz, Iran; 2 Road and Traffic Injury Research Center, Department of Statistics and Epidemiology, Faculty of Health, Tabriz University of Medical Sciences, Tabriz, Iran; 3 Research center of psychiatry and behavioral sciences, Tabriz University of medical sciences, Tabriz, Iran; 4 Tabriz Health services management research center, Tabriz University of Medical Sciences, Tabriz, Iran; 5 Liver and gastrointestinal disease research center, Tabriz University of Medical Sciences, Tabriz, Iran; Hospital de Clinicas de Porto Alegre, BRAZIL

## Abstract

**Background:**

This population-based study aimed at investigating the prevalence and associated factors of prehypertension/hypertension in Iran.

**Methods and Findings:**

The data (n = 2818) for this study were collected in 2015 as a part of the major Lifestyle Promotion Project (LPP) conducted in East Azerbaijan (urban and regional parts). The data for socio-demographic status, dietary information, and physical activity and anxiety levels were collected through validated questionnaires. Then, physical examination including systolic and diastolic blood pressure (SBP, DBP), body mass index (BMI) and conicity index was performed. First-morning spot urine (SU) sample was collected to assume salt intake. The One-way ANOVA, logistic regression, chi-square test and independent t-test were used for statistical analysis. The prevalence of prehypertension, stage I and stage II hypertension, and overall hypertension was 47.3%, 13.6%, 5.45% and 22.6% respectively. The mean systolic (p = 0.004) and diastolic (p<0.001) blood pressure in men were significantly higher than women. Results of logistic regression analysis showed that in both sexes, family history of hypertension, obesity, abdominal obesity, anxiety and having high levels of sodium intake were associated with high blood pressure (p< 0.05). Additionally, 45.8% of the hypertensive patients were aware of their disease, 10.0% of the aware patients, and 44.5% of everyone with high blood pressure were receiving antihypertensive medication.

**Conclusions:**

Our data showed that prehypertension/hypertension is a major health problem in Iran. Focusing on identifying risk factors to hypertension, regular drug intake, good nutrition, physical activity, and changing lifestyles of patients with hypertension are essential.

## Introduction

High blood pressure (BP) is the leading and most important modifiable risk factor for cardiovascular disease (CVD) [1]. Hypertension (HTN) is a global public-health problem, a condition in which blood vessels have persistently raised pressure. It could lead to heart attacks, stroke, kidney failure, blindness, rupture of blood vessels and cognitive impairment [2–4]. The number of adults with hypertension in 2025 is predicted to increase to a total of 1.56 billion [5]. Developing countries are increasingly faced with the double burden of hypertension and other cardiovascular diseases [6, 7]. Previous studies in Iran showed that the prevalence of hypertension in the over 17-year-old population was more than 20% [8, 9].

Along with hypertension, over the last years, the pre-hypertension rates increased worldwide. Cases of pre-hypertension are at higher risk of hypertension compared to those with normal blood pressure [10]. Prehypertension progresses to clinical hypertension at a rate of 19% over 4 years [11] and is also associated with increased risk of CVD [12].

Several risk factors are well-recognized worldwide as contributors to the increase in blood pressure [10]. Rapid population, social, and economic changes in the past decade in Iran have lead to an increasing trend in the prevalence of many CVD risk factors.

There are no recent data available for prevalence and associated factors of prehypertension and hypertension in urban and regional areas of Iran. Data about the prevalence of HTN, mean levels of SBP and DBP and concomitant risk factors can be helpful in planning preventive strategies. This paper presents the first phase of a comprehensive community-based intervention program for prevention and control of non-communicable diseases (NCDs) and their risk factors in Iran (LPP study). This study intends to generate relevant information that helps to understand the patterns of high BP in populations where the prevalence of obesity and other NCDs risk factors is growing rapidly.

## Methods

The data for this study were collected in 2015 as a part of the major Lifestyle Promotion Project (LPP) [13], a longitudinal intervention study to access the lifestyle intervention program conducted in the districts of East Azerbaijan (urban and regional parts), one of the largest provinces of Iran. This study conducted by probability proportional to size (PPS) multistage stratified cluster sampling through which 150 clusters selected. In PPS sampling, the selection probability for each element is set to be proportional to its size measure. The sampling frame of this study was based on the postal code frame of the national post office, which is updated yearly. The clusters were selected in this system based on postal code. Each address in this system was summarized in a 10-digit postal code number. In urban areas, clusters comprise one to several blocks or parts of blocks. Blocks were usually attached buildings. After determining the cluster start point, enrollment and data collection was started. In each cluster, 20 participants (15–65 years) were enrolled (3000 participants). This began from the household at the cluster start point and continued toward the other households until the required number of participants enrolled. Consecutive households were selected based on the geographical location of buildings to the right-hand side of each building. Research survey and examination teams visited households, according to previously arranged appointments. The large sample size and the sampling design and framework mean that the sample was representative of the study population.

The ethical approval for this study was obtained from Ethic Committee of Tabriz University of Medical Sciences (registration number: 1394.383). All procedures performed in this study were in accordance with the ethical standards of the Ethics Committee of Tabriz University of Medical Science and with the 1964 Helsinki declaration and its later amendments or comparable ethical standards. Also, a written informed consent was obtained from all individual participants included in the study.

Exclusion of incomplete questionnaire yielded 2818 final sample, subjected to statistical analysis. The final sample consists of 1370 and 1448 residents of the capital city (Tabriz) and regional areas (including Marand, Mianeh, Varzegan, Khodafarin, Bonab, Osku and Ilkhichi) respectively.

Data was collected by four questionnaires through face-to-face interviews by a team of trained health professionals. Research survey and examination teams visited households according to previously arranged appointments. The first questionnaire included questions regarding the socio-demographic characteristics, including age, gender, educational level, marriage status, employment status, family size and residential area. The second questionnaire was the short food frequency questionnaire. This questionnaire was validated during the project. The third questionnaire was International Physical Activity Questionnaire (IPAQ) and the validity of the translated form of this questionnaire was tested in the previous study on Iranian subjects [14]. Physical activity levels were also classified into three categories: inactive, minimally active and health-enhancing physically active, according to the scoring system provided by the IPAQ [15]. The fourth questionnaire was the Generalized Anxiety Disorder 7-item scale (GAD-7) used as a brief clinical measure of generalized anxiety disorder. The GAD- 7 is a seven-item standardized tool that measures the severity of general anxiety with increasing scores indicating increasing severity of anxiety. Scores of 5, 10, and 15 specify mild, moderate, and severe anxiety, respectively [16].

### Anthropometric measurements

BMI was calculated from height and weight data as kg/m^2^. Overweight was defined as BMI≥25 and obesity was defined as BMI≥30 kg/m^2^. Body-weight was measured to the nearest 0.1 kg on a Seca digital weighing scale, and height was measured to the nearest 0.1 cm, with bare feet using a stadiometer. Waist circumferences (cm) were measured in duplicate with an anthropometric tape while the subjects were wearing light clothing. Waist circumference was measured at the minimum circumference between the iliac crest and the rib cage and conicity index was calculated as follow: Conicity index = waist circumference in meters/ (0.109*square root of weight in kg/height in meters).

### Blood pressure measurement

The blood pressure was measured twice in each arm in the sitting position after a 5 min rest, by a trained nurse using a mercury sphygmomanometer (Richter, Germany). Blood pressure was measured twice on the same day with an interval of 3 min. After two measurements, if there was a difference of more than 10 mmHg in systolic readings and/or 5 mmHg in diastolic readings, blood pressure was measured a third time and the two readings with the least difference were recorded. Average systolic and diastolic readings in these two measurements were considered for systolic and diastolic blood pressure. Prehypertension was defined as having either a SBP of 120 to 139 mmHg and/or DBP of 80 to 89 mmHg, according to JNC7, in persons who were not on antihypertensive medication [17]. Hypertension is defined as systolic blood pressure ≥ 140 and/or diastolic blood pressure ≥ 90 mmHg or current use of antihypertensive medication for management of hypertension, at the time of interview.

### Salt intake

The first-morning spot urine (SU) sample was collected from first voided morning urine. The sodium concentration was measured by an ion-selective electrode method (Modular DPE chemistry; Roche Diagnostics, Mannheim, Germany). Creatinine levels were measured by the Jaffe reaction (Kinetic colorimetric assay; Roche Diagnostics). Kawasaki’s formula was used to estimate urinary sodium excretion over 24-hr [18].

### Sample size

For baseline data collection, based on predicted prevalence rates of different risk factors in subjects 15–64 years of age, confidence interval of 95%, study power of 80%, attrition rate of 20%, design effect of 1.5% for five age groups and two sexes, sample size was calculated to be 1500. However, 3000 subjects involved because of the wide scope of the project and variety of the measured variables.

### Statistical analysis

SPSS v18 statistical computer software was used for all statistical analyses. Means and standard deviations (SDs) were calculated for continuous variables, and proportions were calculated for categorical variables. Between-group comparisons were made by the independent *t-* test and chi-square test. The binary logistic regression models were used to examine the relationships between prehypertension/hypertension (as categorical variables) and associated factors by adjusting for covariates. Covariates for regression analyses included age, marriage, residency area, employment, education, smoking and physical activity status, family history of hypertension, anxiety, energy and salt intake. For each of the categorical variables, the first group was used as the reference category. All analyses were performed for men and women, and urban and regional areas separately. Subjects were subdivided into tertiles according to their salt intake and hypertension rate was compared in different tertiles by chi-square test. A significance level of 0.05 was used.

## Results

Table 1 represents general characteristics of participants and the prevalence of prehypertension and hypertension in urban and regional areas. The mean age and BMI of the participants were 39.71±12.91 years and 26.99±5.26 kg/m^2^ respectively. Regarding the place of residence, 1370 (48.6%) and 1448 (51.4%) participants were living in urban and regional areas respectively. The rates of age, marital, occupational, educational and physical activity status, smoking habit, overweight, obesity, abdominal obesity, prehypertension and hypertension were significantly different in urban and regional areas (p<0.05). The prevalence of overweight, obesity and abdominal obesity was significantly higher in urban areas (p<0.05); however, health enhancing physical activity and smoking was significantly more prevalent in the regional areas (p<0.05). The prevalence of prehypertension (urban = 48.9% vs. regional = 45.8%) and hypertension (urban = 24.7% vs. regional = 20.5%) was significantly higher in urban areas (p = 0.001).

**Table 1:** General characteristics of participants and the prevalence of prehypertension and hypertension in urban and regional areas.

	Urban (n = 1370)	regional (n = 1448)	Total (n = 2818)
Age, % (n)*			
• 15–25	5.1 (71)	8.5 (123)	6.8 (191)
• 26–35	18.7 (257)	22.2 (322)	20.5 (578)
• 36–45	32 (439)	28.4 (412)	30.2 (851)
• 46–55	24 (330)	23.2 (335)	23.8 (671)
• 56–65	19.8 (272)	17.7 (256)	18.7 (527)
Marital status, % (n)*			
• Married	89.1 (1220)	85.5 (1239)	87.1 (2454)
Occupational status, % (n)*			
• Employed or self employed	39.2 (537)	42.4 (614)	40.9 (1153)
• Student	5.6 (77)	6.7 (97)	6.2 (175)
• Unemployed	55.2 (756)	50.9 (737)	52.9 (1490)
Educational status, % (n)*			
• Illiterate	11.2 (153)	14.8 (214)	13.0 (366)
• Under graduate	67.1 (920)	71.2 (1031)	69.2 (1950)
• College	21.7 (279)	14.1 (204)	17.8 (501)
Smoking habit, % (n)*			
• yes	9.5 (130)	12.7 (184)	11.1 (313)
• Occasionally	1.3 (18)	2.0 (28)	1.7 (48)
• No	89.1 (1221)	85.4 (1236)	87.2 (2457)
Physical activity, % (n)*			
• Inactive	43.3 (593)	18.1 (262)	30.3 (854)
• Minimally active	34.8 (477)	29.2 (423)	31.9 (899)
• Health enhancing activity	21.9 (300)	52.7 (763)	37.7 (1062)
Prevalence of overweight, % (n)*	41.6 (570)	37.7 (546)	39.6 (1116)
Prevalence of obesity, % (n)*	25.7 (352)	22.4 324)	24.0 (676)
Prevalence of abdominal obesity, % (n)*	76.3 (1045)	74.1 (1074)	75.2 (2119)
Prevalence of prehypertension, % (n)*	48.9 (670)	45.8 (663)	47.3 (1333)
Prevalence of hypertension, %(n)*	24.7 (339)	20.5 (297)	22.6 (636)

*(p<0.05), differences tested by chi-square test

The prevalence of prehypertension and hypertension stratified by age and sex is presented in Table 2. The prevalence of prehypertension, stage I and stage II hypertension, and overall hypertension was 47.3%, 13.6%, 5.45% and 22.6% respectively. The mean systolic (119.44±15.37 vs. 117.59±17.67 mmHg) and diastolic (77.92±10.51 vs. 76.33±11.66 mmHg) blood pressure in men were significantly higher than women. The prevalence of hypertension was higher in middle-aged adults compared to younger ones. The overall prevalence of hypertension was 38.6% in men and 53.6% in women in the 55–64 year age group.

**Table 2:** The prevalence of prehypertension and hypertension by age and sex.

Variables	Men (n = 1368)	Women (n = 1450)	P value
Systolic blood pressure,mmHg, (mean±SD)^†^	119.44±15.37	117.59±17.67	0.004
• 15–25	111.82±12.00	107.37±13.33	0.02
• 26–35	114.42±11.61	110.05±12.90	<0.001
• 36–45	116.82±13.21	113.15±15.07	<0.001
• 46–55	120.62±14.96	120.37±17.34	0.84
• 56–65	125.52±17.74	128.81±18.09	0.042
P-value*	<0.001	<0.001	
Diastolic blood pressure,mmHg, (mean±SD)^†^	77.92±10.51	76.33±11.66	<0.001
• 15–25	72.56±8.72	69.91±10.36	0.049
• 26–35	75.36±9.90	71.71±10.21	<0.001
• 36–45	76.78±9.75	74.14±10.19	<0.001
• 46–55	79.44±10.71	77.99±11.71	0.11
• 56–65	80.81±10.84	83.02±11.69	0.031
P-value*	<0.001	<0.001	
Prevalence of prehypertension, %^‡^	52.9	41.8	<0.001
• 15–25	47.8	18.6	<0.001
• 26–35	56.8	40.4	<0.001
• 36–45	55.3	42.7	0.003
• 46–55	53.4	43.6	0.002
• 56–65	50.6	47.2	0.37
P-value	0.26	0.84	
Prevalence of Stage 1 Hypertension, %^‡^	13.3	13.9	<0.001
• 15–25	3.5	8.5	<0.001
• 26–35	5.5	4.2	<0.001
• 36–45	8.1	8.0	<0.001
• 46–55	16.4	15.2	<0.001
• 56–65	22.0	29.8	<0.001
P-value	<0.001	<0.001	
Prevalence of Stage 2 Hypertension, %^‡^	5.1	5.8	<0.001
• 15–25	0.0	0.0	-
• 26–35	2.1	1.4	<0.001
• 36–45	3.7	2.2	<0.001
• 46–55	4.2	7.8	<0.001
• 56–65	10.0	12.5	<0.001
P-value	<0.001	<0.001	
Prevalence of total hypertension, %^‡^	21.1	24.3	<0.001
• 15–25	3.5	10.2	<0.001
• 26–35	7.6	5.6	<0.001
• 36–45	12.1	12.3	<0.001
• 46–55	22.9	29.6	<0.001
• 56–65	38.6	53.6	<0.001
P-value	<0.001	<0.001	
Prevalence of hypertension awareness, %^‡^	38.2	53.4	<0.001
• 15–25	0.0	16.7	<0.001
• 26–35	0.0	0.0	-
• 36–45	21.4	32.7	<0.001
• 46–55	32.8	55.0	<0.001
• 56–65	52.1	63.9	<0.001
P-value	<0.001	<0.001	

^†^Differences tested by unpaired Student’s t-test

^‡^Differences tested by chi-square test, One-way ANOVA

The prevalence of prehypertension (52.9% vs. 41.8%) was significantly higher in men compared to women; however, women presented higher prevalence of total hypertension (24.3%vs.21.1%), stage I hypertension (13.9% vs.13.3%) and stage II hypertension (5.8% vs.5.1%) than men. In addition, 45.8% of the hypertensive patients were aware of their disease, 10.0% of the aware patients, and 44.5% of everyone with high blood pressure were receiving antihypertensive medication. In hypertensive patients receiving antihypertensive medication, 30.5% had controlled hypertension; however, in hypertensive subjects without receiving any medication, 2.7% had controlled hypertension. Awareness of being hypertensive was significantly higher in women.

Odds ratios of prehypertension and hypertension for demographic, socio-economic and lifestyle factors were presented in Tables 3 and 4. Results of logistic regression analysis showed that in both sexes, the risk of prehypertension and hypertension was higher in middle-aged adults than in younger ones. In men and women, family history of hypertension, obesity, abdominal obesity, anxiety and having high levels of salt intake were associated with high blood pressure. Regional residents and subjects minimally active or having health enhancing activity were less likely to have prehypertension and hypertension. Marriage, employment, smoking habit and being overweight were not associated with hypertension. Moreover, being overweight was a risk factor for prehypertension in the adjusted model in men and women. Among men and women, higher anxiety was associated with higher risk of prehypertension and hypertension. In the fully adjusted model, the associations between hypertension and education status and anxiety in men, and marriage and employment in both sexes were no longer significant. Additional analysis based on place of residence showed that in urban areas, other than mentioned correlations, the higher level of education was related to the lower rate of hypertension (OR = 0.23, 95% CI = 0.09, 0.59). Additionally, in the regional areas, unemployed subjects were more likely to had hypertension than the students and employed subjects (OR = 1.79, 95% CI = 1.13, 2.83) (Not shown).

**Table 3:** Logistic regression analysis for the association of pre-hypertension and demographic, socio-economic, lifestyle factors.

	Men(n = 1368)		Women(n = 1450)	
Variables	Crude OR (95% CI)	Adjusted OR (95% CI)	Crude OR (95% CI)	Adjusted OR (95% CI)
Age groups				
• 15–25	1.00	1.00	1.00	1.00
• 26–35	1.40 (0.90, 2.18)	2.10 (1.08, 4.06)	2.96 (1.48, 5.95) ***	1.48, 5.95) **
• 36–45	1.33 (0.88, 2.03)	1.87 (0.87, 4.03)	3.19 (1.61, 6.33)***	1.17, 4.65)**
• 46–55	1.21 (0.75, 1.88)	1.82 (1.00, 4.06)*	3.35 (1.68, 6.69) ***	1.08, 3.89) **
• 56–65	1.08 (0.70, 1.69)	2.17 (1.10, 12.8) *	3.99 (1.98, 8.05) ***	1.82 (1.01, 4.63) **
Residential place				
• Urban	1.00	1.00	1.00	1.00
• Rural	0.91 (0.78, 0.97) *	0.54 (0.10, 0.83) **	0.86 (0.55, 0.85) *	0.66 (0.50, 0.88) *
Marital status				
• Single	1.00	1.00	1.00	1.00
• Married	1.79 (1.54, 3.19) *	0.80 (0.46, 1.40)	1.66 (2.05, 2.61) *	1.34 (0.71, 2.53)
Occupational status				
• Employed	1.00	1.00	1.00	1.00
• Student	1.01 (0.69, 1.49)	1.00 (0.52, 90)	0.40 (0.17, 0.93) *	0.30, 3.04)
• Unemployed	1.41 (0.86, 1.94)	0.73 (0.56, 1.38)	1.01 (0.65, 1.47)	0.91 (0.52, 1.60)
Educational status				
• Illiterate	1.00	1.00	1.00	1.00
• Under graduate	0.47 (0.29, 0.67) ***	1.21 (0.67, 2.19)	0.87 (0.65, 1.15)	0.58, 1.32)
• College	0.31 (0.19, 0.54) ***	0.86 (0.271,1.34)	0.91 (0.62, 1.32)	0.93 (0.52, 1.65)
Smoking habit				
• yes	1.00	1.00	1.00	1.00
• Occasionally	1.28 (0.64, 2.57)	2.04 (0.79, 3.41)	1.87 (0.30, 11.62)	0.50, 3.20)
• No	0.97 (0.75, 1.26)	0.85 (0.0.52, 1.46)	0.74 (0.19, 2.88)	0.97 (0.53, 1.96)
Physical activity				
• Inactive	1.00	1.00	1.00	1.00
• Minimally active	0.96 (0.71, 1.29)	0.78 (0.39, 0.84)*	1.00 (0.77, 1.31)	0.61, 0.97) *
• Health enhancing activity	1.00 (0.78, 1.31)	0.66 (0.24, 0.91) *	0.77 (0.53, 1.78)	0.64 (0.32,0.96) *
Family history of hypertension				
• No	1.00	1.00	1.00	1.00
• Yes	1.49 (1.24, 1.93)*	1.25 (1.08, 1.92) *	1.83 (1.42, 2.48) **	1.66 (1.16, 1.94) *
overweight				
• No	1.00	1.00	1.00	1.00
• Yes	1.13 (0.91, 1.427)	1.24 (1.07, 1.75) *	1.25 (1.00, 1.57) *	1.44 (1.00, 2.08)*
Obesity				
• No	1.00		1.00	
• Yes	1.14 (1.07, 2.51) ***	2.16 (1.56, 3.71) **	1.72 (1.33, 2.87) **	1.32 (1.24, 2.94) **
Abdominal obesity				
• No	1.00	1.00	1.00	
• Yes	1.76 (1.35, 3.98) ***	1.18 (1.01, 2.95) **	2.07 (1.55, 4.28) ***	1.50 (1.10, 2.30) *
GAD				
• No anxiety	1.00	1.00	1.00	1.00
• Mild	1.11 (0.62, 1.54)	1.65 (0.72, 3.84)	1.28 (1.09, 1.84) *	0.84, 1.55)
• Moderate	1.24 (0.72, 1.76)	1.74 (0.88, 1.90)	1.39 (1.14, 3.20) **	0.74, 1.81)
• Severe	1.39 (1.13, 2.17) *	1.71 (0.84, 2.19)	1.56 (1.23, 4.41) ***	1.67 (1.14, 2.80) *
Sodium intake				
• Tertile1 (12.5–31.8 g/d)	1.00		1.00	1.00
• Tertile2 (32.5–43 g/d)	1.17 (1.00, 2.33) *	1.43 (1.11, 4.55)*	1.23 (1.07, 3.60) *	1.01, 1.84) *
• Tertile3 (43.5–59.8 g/d)	1.22 (1.07, 7.77) ***	1.99 (1.21, 5.17) **	2.19 (1.57, 12.75) ***	1.55 (1.12, 2.35) **

*p < 0.05

**p < 0.01

***p < 0.001, binary logistic regressions considering the simultaneous effect of all the explanatory variables

**Table 4:** Logistic regression analysis for the association of hypertension and demographic, socio-economic, lifestyle factors.

	Men(n = 1368)		Women(n = 1450)	
Variables	Crude OR (95% CI)	Adjusted OR (95% CI)	Crude OR (95% CI)	Adjusted OR (95% CI)
Age groups				
• 15–25	1.00	1.00	1.00	1.00
• 26–35	1.84 (0.67,5.11)	1.12 (0.18, 7.04)	0.51 (0.19, 1.38)	0.23, 7.38)
• 36–45	3.09 (1.19, 8.01) *	1.35 (0.19, 9.59)	1.24 (0.51,3.04)	0.41, 11.89)
• 46–55	6.82 (2.66,17.47) ***	3.32 (1.46, 23.71)*	3.71 (1.54, 8.91) ***	1.08, 31.33) *
• 56–65	14.35 (5.65, 36.46) ***	7.28 (1.00, 52.72) *	9.95 (4.13,35.41) ***	14.90 (2.71, 81.85) ***
Residential place				
• Urban	1.00	1.00	1.00	1.00
• Rural	0.65 (0.49, 0.84) ***	0.23 (0.16, 0.43) ***	0.79 (0.55, 0.92) *	0.73 (0.44, 0.90) *
Marital status				
• Single	1.00	1.00	1.00	1.00
• Married	5.11 (2.98, 8.78) ***	1.12 (0.36, 3.50)	2.95 (2.04, 4.26) ***	1.01 (0.48, 2.14)
Occupational status				
• Employed	1.00	1.00	1.00	1.00
• Student	0.11 (0.03, 0.36) ***	0.97 (0.15, 6.35)	0.25 (0.05, 0.90) *	0.06, 7.68)
• Unemployed	1.26 (1.05, 2.49) *	0.87 (0.34, 2.23)	1.60 (1.08, 2.70) *	1.19 (0.51, 2.76)
Educational status				
• Illiterate	1.00	1.00	1.00	1.00
• Under graduate	0.33 (0.22, 0.50) ***	1.11 (0.47, 2.60)	1.20 (0.7, 1.99)	0.37, 0.94) *
• College	0.23 (0.14, 0.39) ***	0.64 (0.21,1.88)	0.99 (0.57, 1.74)	0.25 (0.09, 0.66) ***
Smoking habit				
• yes	1.00	1.00	1.00	1.00
• Occasionally	0.47 (0.16, 1.38)	0.85 (0.14, 1.55)	0.66 (0.08, 5.30)	0.24, 1.80)
• No	1.07 (0.78, 1.49)	1.00 (0.76, 1.32)	0.74 (0.19, 2.88)	0.87 (0.59, 2.11)
Physical activity				
• Inactive	1.00	1.00	1.00	1.00
• Minimally active	0.91 (0.63, 1.30)	0.34 (0.16, 0.70) *	0.84 (0.62, 1.13)	0.34, 0.95) *
• Health enhancing activity	0.92 (0.66, 1.22)	0.48 (0.23, 0.91) *	0.95 (0.70, 1.29)	0.58 (0.28, 0.89) *
Family history of hypertension				
• No	1.00	1.00	1.00	1.00
• Yes	1.23 (1.05, 1.60) *	1.19 (1.00, 1.89) *	1.57 (1.22, 2.01) ***	1.61 (1.10, 2.33) *
overweight				
• No	1.00	1.00	1.00	1.00
• Yes	1.35 (0.91, 1.77)	1.36 (0.78, 2.38)	2.84 (0.37, 14.25)	1.36 (0.80, 2.34)
Obesity				
• No	1.00		1.00	
• Yes	2.80 (2.01, 3.89) ***	3.50 (1.79, 6.85) ***	2.75 (2.13, 3.57) ***	2.54 (1.50, 4.31) ***
Abdominal obesity				
• No	1.00	1.00	1.00	
• Yes	2.50 (1.48, 4.22) ***	2.38 (1.11, 6.19) **	2.47 (1.58, 3.87) ***	1.33 (1.04, 3.12) *
GAD				
• No anxiety	1.00	1.00	1.00	1.00
• Mild	1.25 (0.67, 1.99)	1.36 (0.78, 2.35)	1.38 (1.05, 1.81) *	0.90, 2.02)
• Moderate	1.30 (0.78, 2.17)	1.00 (0.42, 2.35)	1.66 (1.09, 4.90) **	0.94, 1.56)
• Severe	1.40 (1.03, 1.91) *	1.09 (0.11, 10.66)	2.94 (1.49, 5.82) ***	2.86 (1.08, 8. 35) *
Sodium intake				
• Tertile1 (12.5–31.8 g/d)	1.00		1.00	1.00
• Tertile2 (32.5–43 g/d)	2.30 (1.79, 19.80) *	1.59 (1.18, 3.77) *	2.01 (1.33, 14.11) *	1.22, 2.96) *
• Tertile3 (43.5–59.8 g/d)	8.75 (1.87, 45.21) ***	2.21 (1.43, 3.44) **	5.45 (1.67, 38.85) ***	2.09 (1.34, 3.25) **

*p < 0.05

**p < 0.01

*** p < 0.001, binary logistic regressions considering the simultaneous effect of all the explanatory variables

**Fig 1:**
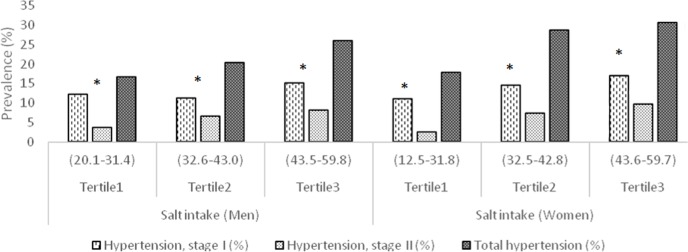
Prevalence of hypertension in men and women in different tertiles of sodium intake.

Fig 1 represents the prevalence of hypertension in men and women in different tertiles of salt intake. In both sexes, an increase in the prevalence of total hypertension, stage I and II hypertension was observed with increasing salt intake. The result of this study showed that almost 100% of subjects had higher sodium intake compared to the recommended value (6 g/day).

## Discussion

Iran is a developing country that is undergoing a rapid epidemiological and nutritional transition. As a result of this rapid transition, hypertension has become one of the emerging public health problems. The results of this study showed that around 47.3% and 22.6% of the participants had prehypertension and hypertension respectively. This rate reflects a significant national hypertension problem. In the first nationwide survey of the prevalence of risk factors for NCDs in the 15–65 y population of Iran in 2007, the prevalence of prehypertension and hypertension in East Azerbaijan was 38.1% and 20.47% respectively [19] which shows that prehypertension/hypertension rates in adults are growing at an alarming rate. The results revealed that nearly half of our population was classified as prehypertensive.

Data from the National Health and Nutrition Examination survey indicated a hypertension prevalence of 29.7% for people 18–74 years of age in the USA [20]. In Cairo (Egypt) almost 60% of the adult population was reported to be hypertensive [21].

The prevalence of prehypertension in the present study (47.3%) was higher in comparison with the prevalence estimates reported in USA (36.3%)[22], China (38.4%)[23] and Turkey (14.5%) [24]. Comparison of data from other countries with the present study suggests that the social and cultural differences, different behavioral and dietary lifestyles, the age span as well as the methodology used have caused different prevalence of hypertension.

A higher percentage of men compared to women were found to exhibit prehypertension (41.8% in women and 52.9% in men). Despite to the higher prevalence of prehypertension in men, the prevalence of hypertension was more alarming among females. A higher prevalence of pre-hypertension in men and hypertension in women has been reported in various studies [9, 24, 25]. Greater BMI among females and their lifestyles were considered as possible factors in previous studies [26, 27]. Oral contraceptives may also increase the risk of hypertension. Meanwhile, menopause can be another factor affecting the prevalence of hypertension in women [27].

In addition, similar to the previous studies, younger men had higher BP than younger women, and older men had lower BP than older women [28]. Based on the results, the prevalence of hypertension rises with increasing age, which is in accordance with the results of other studies [29, 30]. Therefore, planning preventive interventions to control hypertension among older people is essential. In addition, nearly half of individuals (49%) in the 26–45 year age group had prehypertension which indicated a need for prompt intervention for preventing hypertension in this population as a major risk factor for cardiovascular diseases.

Similar to previous studies, increasing age, BMI, low literacy level, low level of physical activity, high level of salt intake and living in urban areas were independent risk factors for prehypertension/hypertension [1, 31].

The results of this study showed that in women, higher education level was correlated with the lower prevalence of hypertension. It seems that compared with people with no formal education, those with a higher education were better informed about hypertension and subsequently had a healthier lifestyle. In addition, based on the results of this study, obesity and abdominal obesity was the strongest modifiable predictor of hypertension, which was consistent with other studies [25, 32]. The relationship between BMI and BP in this study might be potentially confounded by dietary salt intake and physical activity levels, both of which are included in the regression model. As well, abdominal obesity was found to be a significant predictor of hypertension in the both sexes. This is in agreement with studies which showed an important relationship between abdominal obesity and the probability of emerging cardiovascular events [33]. Conicity index was found to be the most important predictor of prehypertension and hypertension in this study. A systematic review and meta-analysis showed that conicity index was a better predictor of hypertension and CVD risk in both sexes in different nationalities [34, 35] and it seems that conicity index is a useful tool for global clinical screening [36].

The urban residency was also associated with increased risk of hypertension in this study. A higher urban prevalence of hypertension was also reported in a multicenter study among elderly people in Bangladesh and India [37, 38]. Increasing urbanization and subsequently changes in levels of risk factors like tobacco smoking, obesity and high levels of physical inactivity possibly contribute to the development of higher BP in Iran.

The results of the current study showed that severe anxiety, especially in women was directly correlated with higher prevalence of hypertension. Our finding is in contrast to the earlier report suggested that no anxiety or mild anxiety was associated with the higher prevalence of hypertension [39, 40]; however, it was consistent with the results of other studies [41, 42]. Epidemiological surveys of mental disorders in Iran reported the rates of mental problems between 20.5% and 21.5% and women had a relative risk of mental disorders of 1.632 compared with men [43]. Some studies have reported that people use eating mechanism as a bodily defensive mechanism against mental problems [44]. Also, lack of self-confidence, depression and anxiety may result in the emergence of the unhealthy condition in lifestyle and subsequently can alter the pattern of NCDs either directly or indirectly [45].

In both sexes, an increase in the prevalence of total hypertension, stage I and II hypertension were observed with increasing salt intake. The result of this study showed that almost 100% of subjects had higher salt intake compared to the recommended value (5g/day).

Dietary salt intake is a known risk factor for hypertension. A large number of studies have been investigated this association, however, the mechanisms by which the increase in salt intake leads to the development of salt-dependent hypertension are not absolutely understood. However, it is recognized that a high-salt diet changes the functioning of the renin-angiotensin system [46]. It is known that a reduction in salt intake will have major beneficial effects on the health of population together with major cost savings [47].

The results of the current study showed that 45.8% of the hypertensive patients were aware of their disease, 10.0% of the aware patients and 44.5% of everyone with high blood pressure were receiving antihypertensive medication. Also, 30.5% of the treated patients, and 2.7% of all hypertensive subjects had controlled hypertension. The proportion of controlled hypertension in this study was significantly lower than the reported data from UK (6%), USA (24%) and France (24%). Awareness of being hypertensive was significantly higher in women. The reasons for the discrepancy between sexes are unknown; however, this can be explained partly by the fact that compared with men, women are more interested in healthcare services and also usually used to express more their unwellness, therefore, more likely to have the higher awareness of the condition [48]. So it seems that it is necessary to implement proper training programs and also offer essential services to health care services by doctors and health experts [49].

The main strength of the present study was a large sample size from different urban and regional areas which provides new data about the prevalence of prehypertension/hypertension in Iran. Moreover, a large number of potential confounders were accessible and several potential confounding factors such as salt intake and anxiety were controlled in the analysis. One of the main limitations of this study is the cross-sectional design of the study which restricts examining causal associations.

In conclusion, our data showed that prehypertension/hypertension is a major health problem in Iran. So, programs to improve the surveillance systems and implementation of community-based screening programs to early detection of the hypertensive cases are needed. Also, improving health literacy to increase public awareness of hypertension, through focusing on regular drug intake, proper nutrition, regular physical activity, anxiety management skills and changing lifestyles of patients with hypertension are essential.
